# Adiponectin Modified BMSCs Alleviate Heart Fibrosis via Inhibition TGF-beta1/Smad in Diabetic Rats

**DOI:** 10.3389/fcell.2021.644160

**Published:** 2021-03-22

**Authors:** Ke Meng, Huabo Cai, Simin Cai, Yucai Hong, Xiaoming Zhang

**Affiliations:** ^1^Department of Anatomy, Sir Run Run Shaw Hospital, School of Medicine, Zhejiang University, Hangzhou, China; ^2^Department of Emergency Medicine, Sir Run Run Shaw Hospital, School of Medicine, Zhejiang University, Hangzhou, China

**Keywords:** diabetic cardiomyopathy, adiponectin, bone marrow mesenchymal stem cells, TGF-beta1, myocardial fibrosis

## Abstract

**Background:** Accumulating evidence suggested that bone marrow mesenchymal stem cells (BMSCs) have therapeutic potential for diabetes and heart diseases. However, the effects of BMSC on reducing myocardial fibrosis need to be optimized. This study aimed to investigate the mechanism of adiponectin (APN) modified BMSCs on myocardial fibrosis in diabetic model *in vivo* and *in vitro*.

**Methods:** The high-fat diet combined with streptozotocin (STZ) injection were used to induced diabetic rat model. H9c2 cells were cultured under a high glucose medium as *in vitro* model. The BMSCs were modified by APN plasmid or APN small interfering RNA (siRNA), then transplanted to the diabetic rats by a single tail-vein injection, or co-cultured with H9c2 cells.

**Results:** We demonstrated that diabetic rats showed typical diabetic symptoms, such as decreased cardiac function, accumulation of pathological lesions and collagen expression. However, these impairments were significantly prevented by the APN modified BMSCs treatment while no effects on APN siRNA modified BMSCs treated diabetic rats. Moreover, we confirmed that APN modified BMSCs could attenuate the expression of TGF-beta1/smad to suppress the myocardial fibrosis in the diabetic rats and high glucose induced H9c2 cells.

**Conclusion:** The present results for the first time showed that APN modified BMSCs exerted protection on cardiac fibrosis via inhibiting TGF-beta1/smad signal pathway in diabetic rats. Our findings suggested that APN modified BMSCs might be a novel and optimal therapy for the diabetic cardiomyopathy in future.

## Introduction

Diabetes mellitus and its complications become a dominant public health problem (American Diabetes Association, 2012). Clinical cases suggested that heart failure incidence was significantly higher in patients with diabetes (Cavender et al., 2015). The studies revealed that hyperglycemia could directly injure cardiomyocytes leading to diabetic cardiomyopathy (DCM) (Seferovic and Paulus, 2015; Jia et al., 2018b). DCM is one of diabetic complications that causes heart failure in patients with diabetes (Wang et al., 2018). The pathogenesis of DCM is complex, and cardiomyocyte fibrosis is the main distinct pathological features of DCM (Chang et al., 2016; Faramoushi et al., 2016). Biopsies from diabetic patients with heart failure indicated that cardiac fibrosis might play a vital role in the DCM (Falcao-Pires et al., 2011). Cardiac fibrosis contributes to cardiac remodeling and finally leads to the progression of heart failure (Jia et al., 2018a). The transforming growth factor-beta (TGF-β) superfamily serves multiple roles in cell differentiation, proliferation and fibrosis. The TGF-βs have three isoforms (β1, β2, and β3) in mammals (Travis and Sheppard, 2014), and TGF-β1 is implicated in fibrosis (Oruqaj et al., 2015). TGF-β1 over-expressed in myocardium induced stable hypertrophy to heart failure. Upregulation of TGF-β1 was related to fibrosis in diabetic heart (Koitabashi et al., 2011). High glucose could increase TGF-β1 activity and downstream canonical Smad signaling that increased interstitial fibrosis and cellular hypertrophy in the heart of diabetic rats (Bugyei-Twum et al., 2014). Moreover, TGF-β1/Smad may play an important role in fibrogenesis of DCM (Shen et al., 2014).

Bone marrow mesenchymal stem cells (BMSCs) are multipotent stem cells and emerged protection through paracrine related factors and immunomodulatory to host cells (Jing et al., 2014). The scientists found that BMSCs could improve cardiac functions against DCM (Li et al., 2008). However, the effects of BMSCs on reducing cardiac fibrosis still need to be optimized. Interestingly, the adiponectin (APN) that secreted by adipocytes had anti-insulin resistance, anti-atherosclerotic, and anti-inflammatory effects (Wang et al., 2013). Furthermore, APN-knockout mice could enhance carbon tetrachloride-induced liver fibrosis (Nishihara et al., 2006) and renal fibrosis (Tian et al., 2018), while increasing APN expression attenuated liver and renal fibrosis. Therefore, we hypothesized a novel therapeutic effect of the APN modified BMSCs on DCM. The present study aimed to investigate the effects and possible signal pathways involved in APN modified BMSCs on cardiac structural and functional improvement of diabetic models *in vivo* and *in vitro*.

## Materials and Methods

### Ethics Statement

The animal experiments were performed in accordance with the guidelines of the Institutional Animal Care and Use and approved by the Committee of Animal Experiment Center of Zhejiang University (Hangzhou, China). The best efforts have been made to minimize the number and the suffering of the rats.

### Primary BMSCs Culture and Characterization

As our previous study reported (Wang et al., 2016), the primary BMSCs were harvested from the femurs of 2-week-old Sprague-Dawley (SD) female rats that purchased from Shanghai laboratory animal center (Shanghai, China). The rats were anesthetized with sodium pentobarbital (40 mg/kg). The bone marrow cavity was flushed and then cultured in Dulbecco’s modified Eagle’s medium (DMEM, Catalog: 11885-084, Gibco, Invitrogen, United States) with 10% (V/V) fetal bovine serum (Catalog: 10099-141, Gibco, Invitrogen, United States) and a mixture of 1% penicillin and streptomycin (Catalog: 10378016, Invitrogen, United States) and maintained in a tri-gas incubator (Thermo Fisher Scientific, Marietta, OH, United States) composed of 5% CO_2_ and 95% air at 37°C and changed the fresh medium every 2 days.

### Lentivirus Vector Construction and Infection of BMSCs

Recombinant lentivirus vector (LV) containing green fluorescent protein (GFP) as LV5-APN-GFP and LV3-APN-small interfering RNA (siRNA)-GFP, they were designed and constructed by Shanghai GenePharma Technology Co., Ltd. (Shanghai, China), and LV3-vehicle-GFP virus that expresses GFP only was also constructed as a control (Sanber et al., 2015). The target gene APN (Gene Bank accession NM_031012.1) fragment was inserted into the *Not*I/*Bam*HI site on the LV5 vector. The target siRNA against rat APN gene was designed as following: 5′-GGATCTCGTTGTTGGGCTTTA-3′. The lentivirus was determined to be 1 × 10^8^ PFU/ml by using a titration kit, and the lentivirus vector was stored at −80°C until use. BMSCs (5 × 10^5^ cells) were seeded into the 6-well plate, incubated overnight, and then transfected with LV3-GFP, LV5-APN-GFP, and LV3-APN-siRNA-GFP for 72 h as BMSCs/Vehicle, BMSCs/APN+, and BMSCs/APN− separately (Sanber et al., 2015). The multiplicity of infection (MOI) was 100. Transduction efficiency of APN was examined after 48 h by fluorescence microscope (BX-53, Olympus Corp., Tokyo, Japan). The expression of APN was detected by western blot analysis.

### Animal Models Induction

Male SD rats were obtained from Shanghai laboratory animal center (Shanghai, China). The rats were divided into six groups at random: the control group, the DM (diabetic rat) group, the BMSCs/Vehicle group, the BMSCs/APN+ group, and the BMSCs/APN− group (*n* = 10). After 18 h of fast, the rats were given a single intraperitoneal injection of freshly prepared streptozotocin (STZ; 65 mg/kg body weight) (Catalog: S8050, Solarbio, Beijing, China) dissolved in 0.01 M citrate buffer (pH 4.5) for diabetes modeling, while the control rats were intraperitoneally injected with equal amounts of citrate buffer (pH 4.5). A total of 72 h after injection, the fasting blood glucose was determined using an analyzer (Accu-Chek^®^, Roche, Mannheim, Germany). Rats with blood glucose levels above 16.7 mmol/L were considered successful diabetic models for further study (Hasegawa et al., 2010). One week after STZ injection, the 200 μl PBS including 4 × 10^6^ BMSCs, BMSCs/Vehicle, BMSCs/APN+, and BMSCs/APN− were injected into the diabetic rat by a single tail-vein injection as the BMSCs group, the BMSCs/Vehicle group, the BMSCs/APN+ group, and the BMSCs/APN− group, respectively.

### Echocardiography Assay for the Cardiac Function and Metabolic Indexes Analysis of All Groups Rats

Six weeks after transplantation, the cardiac function was estimated through a M9 echocardiography machine (Mindray, Shenzhen, China). We anesthetized all rats with an intraperitoneal sodium pentobarbital (40 mg/kg) and removed the chest hair by a pet razor. A 30-MHz probe was then used to carry out the exam (Scherrer-Crosbie et al., 1998). The left ventricular internal diameter end diastole (LVIDD), left ventricular internal diameter end systole (LVIDs), left ventricular ejection fraction (LVEF), and fractional shortening (FS) were measured by an echocardiography doctor who blinded to all groups. After echocardiography assay, the body weight and fasting blood glucose were recorded on the basis of fasting for 8 h. All rats were anesthetized with an intraperitoneal sodium pentobarbital (40 mg/kg). We removed all rat’s hearts, and recorded heart weight, then for subsequent analysis.

### Masson, Hematoxylin and Eosin and Immunohistochemistry Staining

For pathological analysis, the heart sections were respectively subjected to Masson and hematoxylin and eosin (HE) staining (Wang et al., 2014). The hearts of five animals from each group were removed and fixed in 4% paraformaldehyde, then embedded in paraffin and sliced into sections (8 μm thick). We used HE (Catalog: G1005, Solarbio, Beijing, China), Masson’s trichrome staining (Catalog: G1006, Servicebio, Wuhan, China), and immunohistochemistry staining to determine the left ventricular wall thickness, the collagen area, and the cross-sectional area of the cardiomyocytes. All images of the sections were collected by a light microscope (BX-53, Olympus Corp., Tokyo, Japan), and analyzed with Image-Pro Plus 6.0 software (Media Cybernetics, MD, United States) for positive area measurement of whole vision field in each figure under 400× microscope to analyze the positive area of Masson’s trichrome staining that relative to whole myocardium area (%).

Expression and localization of the target protein were observed by immunohistochemistry methods. The serial heart sections were incubated with primary antibodies overnight at 4°C, including TGF-β1 (Catalog: GB11179, 1:100, Boster Bio, Wuhan, China); Smad2 (Catalog: BA4557, 1:100, Boster Bio, Wuhan, China); Smad3 (Catalog: BA4559, 1:100, Boster Bio, Wuhan, China); Collagen I (Catalog: BA0325, 1:100, Boster Bio, Wuhan, China), and Collagen III (Catalog: BA0326, 1:100, Boster Bio, Wuhan, China). Sections were washing with PBS and subsequently incubated with biotinylated and affinity-purified IgG secondary antibodies at room temperature for 4 h. Images were visualized by a light microscope (BX-53, Olympus Corp., Tokyo, Japan) and analyzed by Image-Pro Plus 6.0 (Media Cybernetics, Maryland, United States) for positive area measurement of whole vision field in each figure under 400× microscope to analyze the positive area of collagen I and collagen III Immunostaining that relative to whole myocardium area (%).

### H9c2 Co-cultured With Modified BMSCs Under High Glucose Medium

Rat cardiomyocyte cell line H9c2 cells were cultured in DMEM medium (Catalog: 11885-084, Gibco, Invitrogen, United States) with 10% (V/V) fetal bovine serum (Catalog: 10099-141, Gibco, Invitrogen, United States), and supplement with 25 mM glucose (control group) or with 50 mM high glucose (DM group), at a humidified atmosphere containing 5% CO_2_ and 95% air. The above BMSCs (5 × 10^5^/well) were seeded in the insert chamber of 6-well 0.4 μm transwell system, then co-cultured with 5 × 10^5^/well H9c2 in DMEM medium with 50 mM high glucose as DM+ BMSCs group, DM+ BMSCs/Vehicle group, DM+ BMSCs/APN+ group, and DM+BMSCs/APN− group, respectively. After 7 d, H9c2 cells were washed twice with PBS, lysed in Western and IP buffer (Catalog: P0013, Beyotime Institute of Biotechnology, Shanghai, China), then stored at −80°C for western blot analysis.

### Western Blot Analysis

Proteins expression of rat’s hearts and co-cultured cells were analyzed by western blot. The BCA protein concentration assay kit was used for protein quantification. Equal amounts of protein were separated using 10% sodium dodecyl sulfate-polyacrylamide gel electrophoresis (SDS-PAGE) and transferred from the gel to nitrocellulose membrane, blocked for 1 h with 5% skimmed milk in Tris-buffered saline containing 0.1% Tween-20 at room temperature. The membranes were incubated overnight at 4°C with the primary antibodies TGF-β1 antibody (Catalog: 3711, 1:1000); Smad2 (Catalog: 5339, 1:1000); phospho-Smad2 (Catalog: 3108, 1:1000); Smad3 (Catalog: 9523, 1:1000), and phospho-Smad3 (Catalog: 9520, 1:1000) purchased from Cell Signaling Technology (Danvers, MA, United States). Actin (Catalog: A2228, 1:5000) was purchased from Sigma-Aldrich (Merck, KGaA, Darmstadt, Germany). After incubation for 16 h, the membranes were washed with TBST and incubated with infrared labeled secondary antibody for 1 h at room temperature and washed three times with TBST. Immunoblotted bands were analyzed by CLx Odyssey infrared imaging system (Li-COR biosciences, United States).

### Statistical Analysis

Values were expressed as mean ± standard deviation (SD). All parameters were statistically performed by one-way analysis of variance (ANOVA).*p* < 0.05 was considered statistically significant.

## Results

### The BMSCs Isolation and APN Modification

**FIGURE 1:**
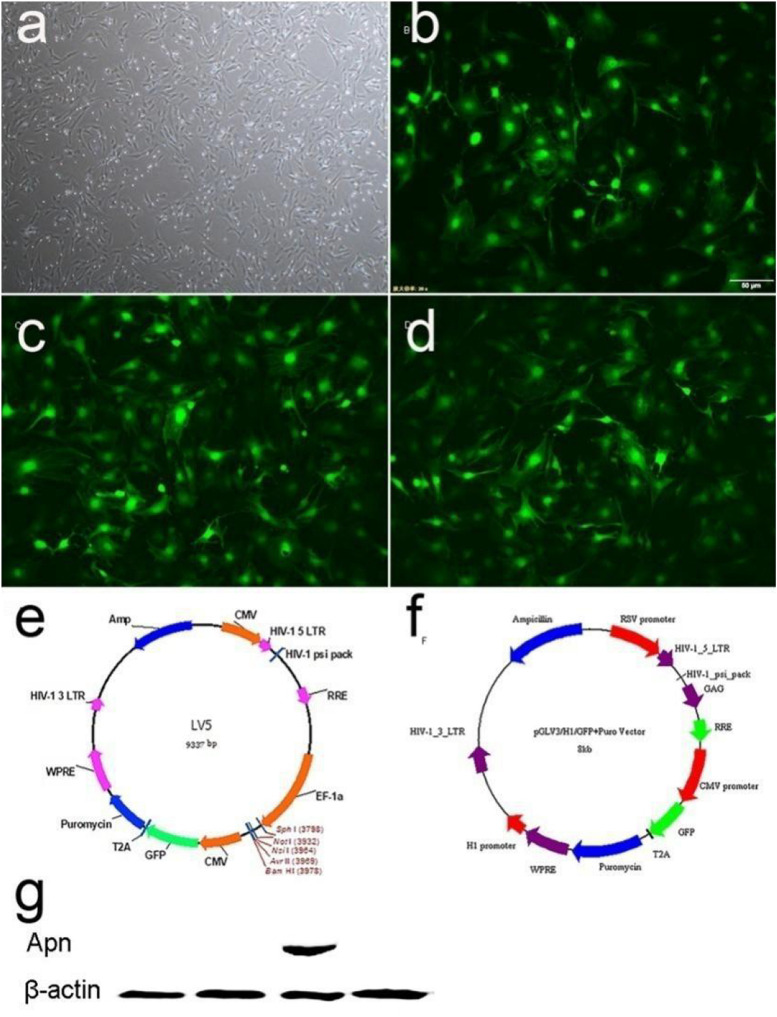
The APN plasmid construction and expression. **(a)** Morphology characterization of cultured BMSCs (light microscopy). **(b)** GFP expression in BMSCs 48 h after transduction of empty vector into BMSCs. **(c)** GFP expression in BMSCs 48 h after transduction of APN into BMSCs. **(d)** GFP expression in BMSCs 48 h after transduction of APN-siRNA into BMSCs. **(e)** Maps of LV5. **(f)** Maps of LV3. **(g)** Western blotting detection of APN protein expression.

The primary BMSCs were isolated and identified as our previous study (Wang et al., 2016). The GFP was used to detect the transfection efficiency of the virus (Figures 1e,f). A total of 48 h after infection, BMSCs were infected by lentivirus with GFP, and normal morphology was showed in three groups by fluorescence microscope (BX-53, Olympus Corp., Tokyo, Japan) (Figures 1a–d). The results indicated over 80% transduction efficiency in BMSCs. A total of 72 h after transfection, the expression of APN was significantly increased in the BMSCs/APN+ group, while almost no detectable levels in the BMSCs, the BMSCs/Vehicle, and the BMSCs/APN− group (Figure 1g).

### The Metabolic Indexes in All Groups

**FIGURE 2:**
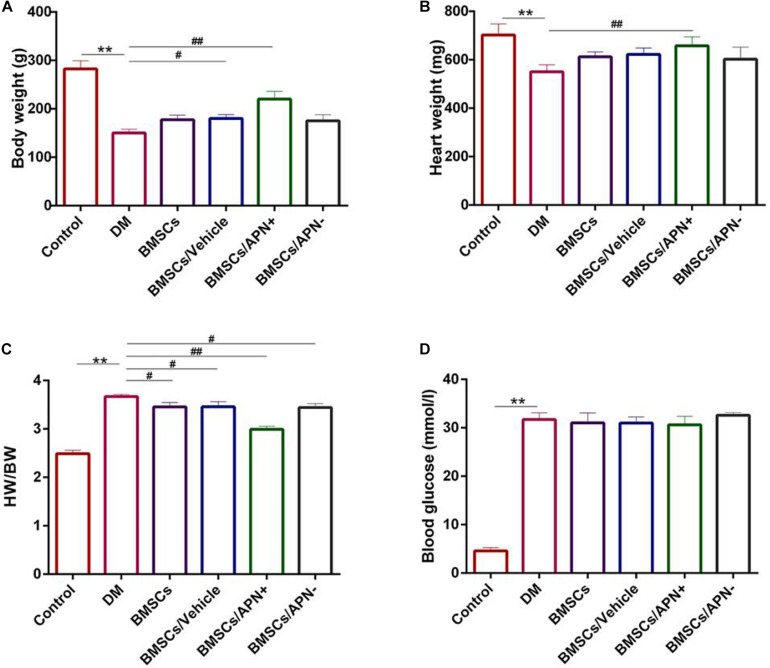
Basic characteristics of experiment groups changes at 6 weeks. **(A)** Body weight (g). **(B)** Heart weight (mg). **(C)** Heart weight to body weight ratio (HW/BW). **(D)** Blood glucose (mmol/l). Date are mean ± SD; *p < 0.05, **p < 0.01, ^#^*P* < 0.05, vs DM, ^##^*P* < 0.01, vs DM.

Six weeks after treatment, the body weight of DM rats was lower than that of the control rats (Figure 2A, *p* < 0.01), which accompanied by the decrease of the heart weight (Figure 2B, *p* < 0.01). There is no remarkable differences in the BMSCs group, the BMSCs/Vehicle group and the BMSCs/APN− group. However, both of body weight and heart weight showed a significant increase in the BMSCs/APN+ group while comparing to the diabetic rats (Figures 2A,B, *p* < 0.01).

The ratio of heart weight to body weight (mg/g) was increased in the DM group and decreased in the BMSCs, the BMSCs/Vehicle, the BMSCs/APN− group, and BMSCs/APN+ group (Figure 2C, *p* < 0.01). After STZ injection, the blood glucose increased while compared to the control rats. There are no significant differences in the rest groups (Figure 2D, *p* > 0.05).

### APN Modified BMSCs Attenuated Diabetes-Induced Cardiac Dysfunction

**FIGURE 3:**
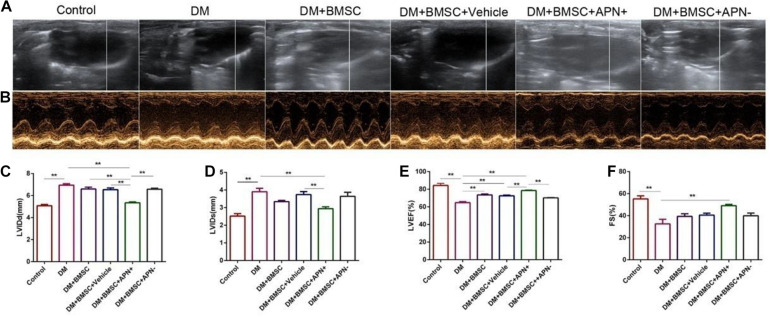
Effects of APN on diabetes-induced LV dysfunction. **(A)** Two-dimensional echocardiograms. **(B)** M-mode echocardiograms. **(C)** Left ventricular internal diameter at end-diastole (LVIDD). **(D)** Left ventricular internal dimension systole (LVIDs). **(E)** LV ejection fraction (LVEF). **(F)** Fractional shortening (FS). Data are mean ± SD; *p < 0.05, **p < 0.01.

The cardiac structure and systolic/diastolic function in all rats were assessed by echocardiogram. Six weeks after treatment, the LVEF ratio was above 70, and the FS ratio was more than 40 in the normal rats. The DM rats exhibited a larger LVIDD/s diameter (*p* < 0.01, Figures 3A–D) and LVEF. However the FS ratio decreased (*p* < 0.01, Figures 3A,E,F) when compared to the control rats. BMSC/APN+ treatment dramatically improved diameter in LVIDD, LVIDs, LVEF, and FS ratio (*p* < 0.01, Figure 3). There is no improvement of LVIDD and LVIDs in rats of BMSCs, BMSCs/Vehicle, and BMSCs/APN− group (Figure 3, *p* > 0.05). Moreover, the LVEF ratio of the BMSCs group and BMSCs/Vehicle group also improved significantly (Figure 3, *p* < 0.01) while the FS ratio of the BMSCs group, the BMSCs/Vehicle group, and the BMSCs/APN− group did not show significant alterations (Figures 3A,F, *p* > 0.05).

### APN Modified BMSCs Improved Pathological Injury of Diabetic Heart

**FIGURE 4:**
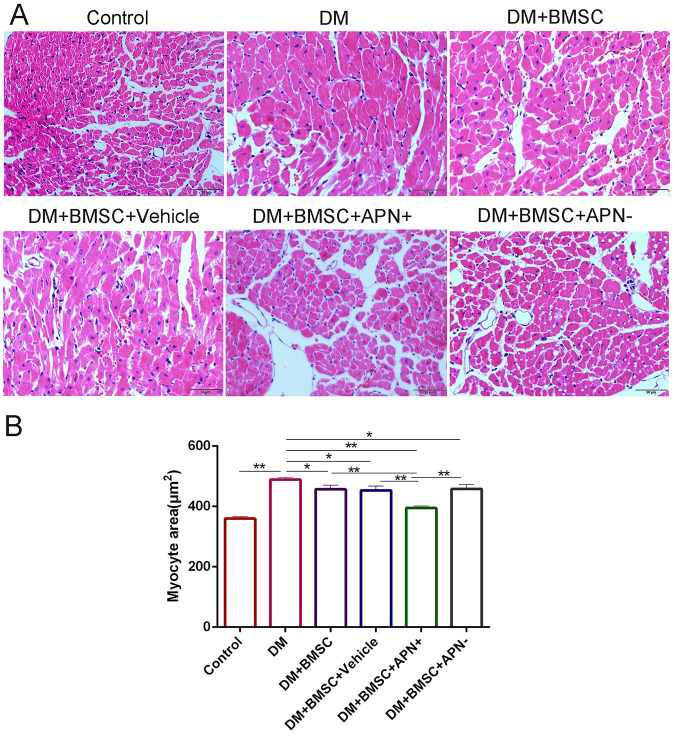
APN attenuated pathological changes in the hearts of diabetic rats. **(A)** Representative micrographs of myocardial tissue sections stained with hematoxylin and eosin (scale bar: 50 μm). **(B)** Quantitative analysis of myocyte size area. Data are mean ± SD; *p < 0.05, **p < 0.01.

We analyzed the pathological changes of myocardial tissue to investigate the benefit of BMSCs/APN+ on diabetes-induced myocardial structural injury. The results demonstrated that diabetic heart exhibited cardiomyocyte hypertrophy, irregular myocardial arrangements, increased interstitial spaces, and myofibrillar discontinuation (Figure 4A). The normal control heart had regular myocardial structures and clear visible nuclei. Furthermore, BMSCs/APN+ group rats could improve these pathological changes. Cross-sections of left ventricular myocardial were examined at a magnification of 400× (Figure 4B). The myocyte areas of LV were significantly increased in the DM rats, while compared with the control rats (Figure 4B, *p* < 0.01). The results indicated that transplanted BMSCs could reduce the increasing myocyte size, especially in the BMSCs/APN+ treatment groups (Figure 4C, *p* < 0.01).

### APN Modified BMSCs Improved Diabetes-Induced Myocardial Fibrosis

**FIGURE 5:**
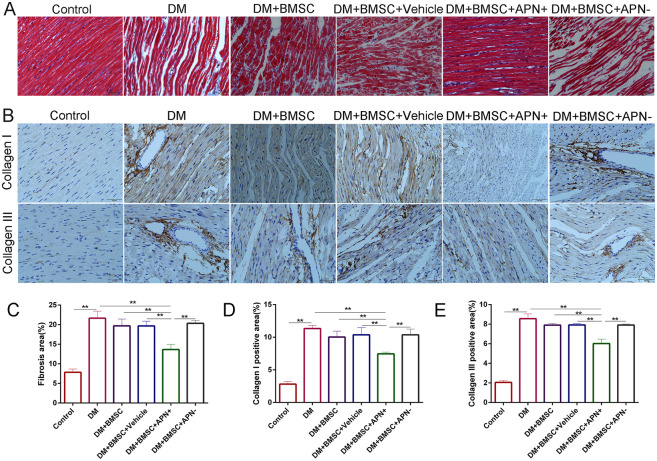
Effect of APN on collagen deposition. **(A)** Representative Masson’s trichrome staining. **(B)** Immunostaining of collagen I and collagen III. **(C)** Quantitative analysis of Masson’s trichrome staining. **(D)** Immunohistochemistry analysis of collagens I. **(E)** Immunohistochemistry analysis of collagens III. Data are mean ± SD; *p < 0.05, **p < 0.01.

Myocardial fibrosis is the major pathological lesion of DCM. Masson’s trichrome staining of the heart showed certain irregularity of the myocardial fibrosis and increased collagen accumulation in the diabetes rats when compared with the normal heart. However, BMSCs/APN+ treated rats showed a remarked improvement for reduction collagen accumulation when compared to the diabetes rats (Figures 5A,C, *p* < 0.01). The collagen I and III, as cardiac fibrotic markers, were augmented under diabetic conditions and BMSCs/APN+ could reduce collagens I and III expression (Figure 5, *p* < 0.01). There is no significant differences of collagens I and III expression in the rest groups (Figure 5, *p* > 0.05). These results demonstrated that BMSCs/APN+ could attenuate myocardial fibrosis and improve the collagen deposition in diabetic hearts.

### APN Modified BMSCs Alleviates Diabetes-Induced TGF-β1/Smad 2/3 Signaling Pathway Activation

**FIGURE 6:**
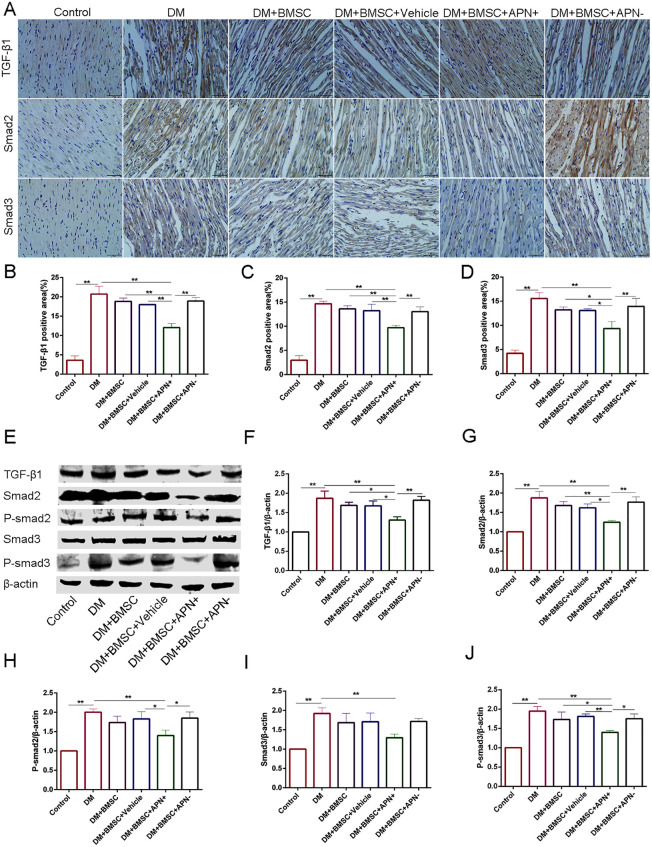
The expression of TGF-β1/Smad 2,3 in all groups rats. **(A)** Immunostaining of TGF-β1, Smad2 and Smad3. **(B)** Quantitative analysis of TGF-β1. **(C)** Quantitative analysis of Smad2. **(D)** Quantitative analysis of Smad3. **(E)** Representative western blot: TGF-β1, Smad2, P-smad2, Smad3, and P-smad3. **(F)** The western blot assay of TGF-β1. **(G)** The western blot assay of Smad2. **(H)** The western blot assay of P-smad2. **(I)** The western blot assay of Smad3. **(J)** The western blot assay of P-smad3. Data are mean ± SD; *p < 0.05, **p < 0.01.

To verify the possible signaling pathway involved in myocardial fibrosis of diabetic heat, we analyzed the expression of TGF-β/Smad2/3 by immunohistochemistry. The results showed their expression in the nucleus (Figure 6A) and the TGF-β1/Smad2/3 protein increased in diabetic hearts (Figure 6, *p* < 0.01), while the BMSCs/APN+ treatment can reverse the these upregulation (Figure 6, *p* < 0.01). However, there is no detectable difference in the rest three groups (Figure 6, *p* > 0.05). The western blot analysis demonstrated that the TGF-β1/Smad2/3 protein had significant reduction in the BMSCs/APN+ group (Figure 6, *p* < 0.01), and no marked alteration in the BMSCs group, the BMSCs/Vehicle group, and the BMSCs/APN− group as an assumption (Figure 6, *p* > 0.05). We also detected the expression of p-smad 2/3 protein, and found it phosphorylation was positively stressed in the BMSCs/APN+ diabetic rats (Figure 6, *p* < 0.01), but there is no significant improvement in the rest three groups (Figure 6, *p* > 0.05). These results indicated that BMSCs/APN+ could inhibit cellular TGF-β1/Smad2/3 related signal pathways, which may alleviate diabetes-induced myocardial fibrosis in diabetic rats.

### BMSCs/APN+ Co-cultured With H9c2 Inhibited High Glucose Induced TGF-β1/Smad2/3 Activation

**FIGURE 7:**
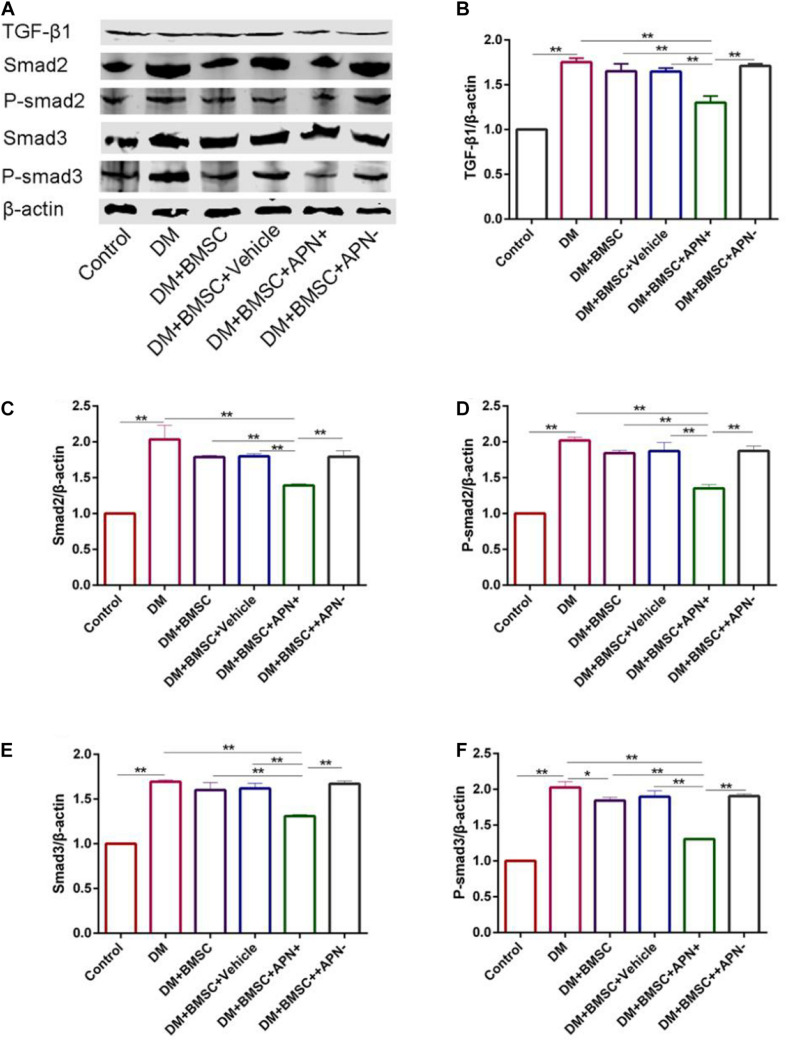
The expression of TGF-β1/Smad 2,3 in high glucose cultured H9c2 cells. **(A)** Representative western blot: TGF-β1, Smad2, p-smad2, Smad3, and p-smad3. **(B)** The western blot assay of TGF-β1. **(C)** The western blot assay of Smad2. **(D)** The western blot assay of p-smad2. **(E)** The western blot assay of Smad3. **(F)** The western blot assay of p-smad3. Data are mean ± SD; *p < 0.05, **p < 0.01.

To clarify the role of BMSCs/APN+ treatment in collagen deposition *in vitro*, we cultured H9c2 in normal glucose (25 mM) as the control group and high glucose (50 mM) as the DM group. H9c2 in high glucose co-cultured with the BMSCs, the BMSCs/Vehicle, the BMSCs/APN+, and the BMSCs/APN− for 7 days, respectively. The western blot analysis showed that H9c2 cultured in high glucose significantly increased the TGF-β1 Smad2, P-smad2, Smad3, and P-smad3 when compared with the normal control group (Figure 7, *p* < 0.01). BMSCs/APN+ could inhibit the expression of TGF-β1/Smad2/3 (Figure 7, *p* < 0.01). There were no significantly altered in the BMSCs, the BMSCs/Vehicle, and BMSCs/APN− treated H9c2 cells while compared with the high glucose cultured cells (Figure 7, *p* > 0.05). The present results demonstrated that BMSCs/APN+ could alleviate high glucose-induced TGF-β1/Smad2/3 activation.

## Discussion

Cardiac fibrosis is one of the most common pathological lesions of DCM, ultimately leading to heart failure (Huynh et al., 2014). Therefore, reducing the excessive proliferation of collagen in the myocardium is necessary for preventing DCM and heart failure (Talior-Volodarsky et al., 2012). The present study for the first time suggested that APN modified BMSCs could improve fibrosis in diabetic heart *in vitro* and *in vivo*. During a 6-week follow-up period, we found a significant increase of collagen fibers and pathological changes in diabetic heart. The expression of collagen I and III also increased significantly in diabetic myocardium. The echocardiographic results showed that sustained increasing collagen fibers in the myocardium might damage the cardiac function. Interestingly, a tail-vein injection of BMSC/APN+ caused significant improvement of body weight, cardiac function, collagen accumulation, and attenuated the pathological lesions in diabetic hearts.

Myocardial fibrosis is a chronic and progressive process characterized by an excessive accumulation of collagen fibers in diabetic hearts (Luo et al., 2013). Cardiac fibrosis contributes to cardiac remodeling, and finally leads to the progression of heart failure. Under diabetes conditions, myocardial fibrosis is regulated by various factors. The previous study revealed that TGF-β1 is the most important one (Sui et al., 2015). The studies of diabetic animals revealed that increased TGF-β1 was associated with cardiac fibrosis. Moreover, TGF-β inhibition can attenuate cardiac fibrosis in animal models (Avila et al., 2014). The scientists found that high glucose could increase TGF-β1 activity and downstream canonical Smad signaling that increased interstitial fibrosis and cellular hypertrophy in diabetic hearts (Shen et al., 2014). Activated TGF-β1 binds to the TGF-β1 receptor, it will allow the phosphorylation of the Smad 2/3 proteins. These Smads can be directly activated through phosphorylation by the TGF-β type I receptor kinase. Smad3 has been recognized as a key mediator of TGF-β-induced pro-fibrotic outcomes. Both Smad2 and Smad3 have been implicated in ECM production and tissue fibrosis (Yuan et al., 2013). Subsequently, these Smads complexes can be translocated into the nucleus and then regulate the transcription of the target genes, such as pro-fibrotic genes, that play crucial roles in the development of myocardial fibrosis (Zhang et al., 2014).

In the present study, the immunohistochemistry and western blot assay results demonstrated that the expression of TGF-β1, Smad2/3, and p-Smad2/3 protein in the diabetic hearts tended to increase. Furthermore, BMSCs/APN+ treatment could decrease Smad2/3 phosphorylation, be consistent with the improvement of collagen formation and cardiac function while BMSCs/APN− have no effects on cardiac fibrosis, functional recovery in the diabetic hearts. Under the condition of long-term high glucose stimulation, the expression of TGF-β1 in cardiomyocytes increases, which may induce fibroblasts to synthesize a large amount of collagen, promote fibroblast proliferation, and transform into myofibroblasts (Zhang et al., 2014). The APN modified BMSCs may reduce myofibrillar disorder and inhibit the proliferation and transformation of cardiac fibroblasts, as well as the interaction between fibroblasts and cardiomyocytes via TGF-β1/Smad signal pathway.

H9c2 cell line showed an undifferentiated phenotype, which was similar to the physiological characteristics of normal cardiomyocytes in morphology, biochemistry and other characteristics, and was easy to obtain, passable and stable, which makes up for the poor repeatability of primary cardiomyocytes. H9c2 cardiomyocytes also have some limitations when used in isolated heart models. The present data demonstrated that high glucose increased expression of TGF-β1 and p-Smad2/3 in H9c2 cells while co-cultured with the BMSCs/APN+ could reduce the expression of TGF-β1 and p-Smad2/3. These results were consistent with the animal studies.

## Conclusion

In summary, the present data demonstrate a protective role of BMSCs/APN+ on diabetic hearts. Interestingly, high glucose increased TGF-β1, p-Smad2/3, and collagen fibers deposition while BMSCs/APN+ could suppress these trend and improve functional recovery in the hearts of diabetic rats. Therefore, the present study provided a novel strategy for the treatment of DCM in patients with diabetes.

## Data Availability Statement

The raw data supporting the conclusions of this article will be made available by the authors, without undue reservation.

## Ethics Statement

The animal study was reviewed and approved by the Committee of Animal Experiment Center of Zhejiang University (Hangzhou, China).

## Author Contributions

KM performed the cells culture and animal model. KM and HC contributed to writing the manuscript and the data analysis. SC assisted with the experiments. YH and XZ conceived the study and contributed to the manuscript preparation. All authors read and approved the final version of the manuscript.

## Conflict of Interest

The authors declare that the research was conducted in the absence of any commercial or financial relationships that could be construed as a potential conflict of interest.

## Abbreviations

APN: adiponectin
BMSCs: bone marrow mesenchymal stem cells
DCM: diabetic cardiomyopathy
DMEM: Dulbecco’s modified Eagle’s medium
FS: fractional shortening
GFP: green fluorescent protein
HE: hematoxylin and eosin
LV: lentivirus vector
LVIDD: left ventricular internal diameter end diastole
LVIDs: left ventricular internal diameter end systole
LVEF: left ventricular ejection fraction
MOI: multiplicity of infection
SD: Sprague-Dawley
SDS-PAGE: sodium dodecyl sulfate-polyacrylamide gel electrophoresis
siRNA: small interfering RNA
STZ: streptozotocin
TGF-β: transforming growth factor-beta.
