# Corrigendum: Adiponectin modified BMSCs alleviate heart fibrosis via inhibition TGF-beta1/smad in diabetic rats

**DOI:** 10.3389/fcell.2023.997572

**Published:** 2023-05-11

**Authors:** Ke Meng, Huabo Cai, Simin Cai, Yucai Hong, Xiaoming Zhang

**Affiliations:** ^1^ Department of Anatomy, Sir Run Run Shaw Hospital, School of Medicine, Zhejiang University, Hangzhou, China; ^2^ Department of Emergency Medicine, Sir Run Run Shaw Hospital, School of Medicine, Zhejiang University, Hangzhou, China

**Keywords:** diabetic cardiomyopathy, adiponectin, bone marrow mesenchymal stem cells, TGF-beta1, myocardial fibrosis

In the published article, there was an error in Figures 4–6 as published. The tissue photograph of Figure 4A DM + BMSC + APN+, Figure 5A DM + BMSC + APN-, Figure 5B collagen I DM + BMSC &DM + BMSC + APN-, Figure 6A DM + BMSC + APN- are wrong**.** The corrected Figures 4–6 appear below.

**FIGURE 4:**
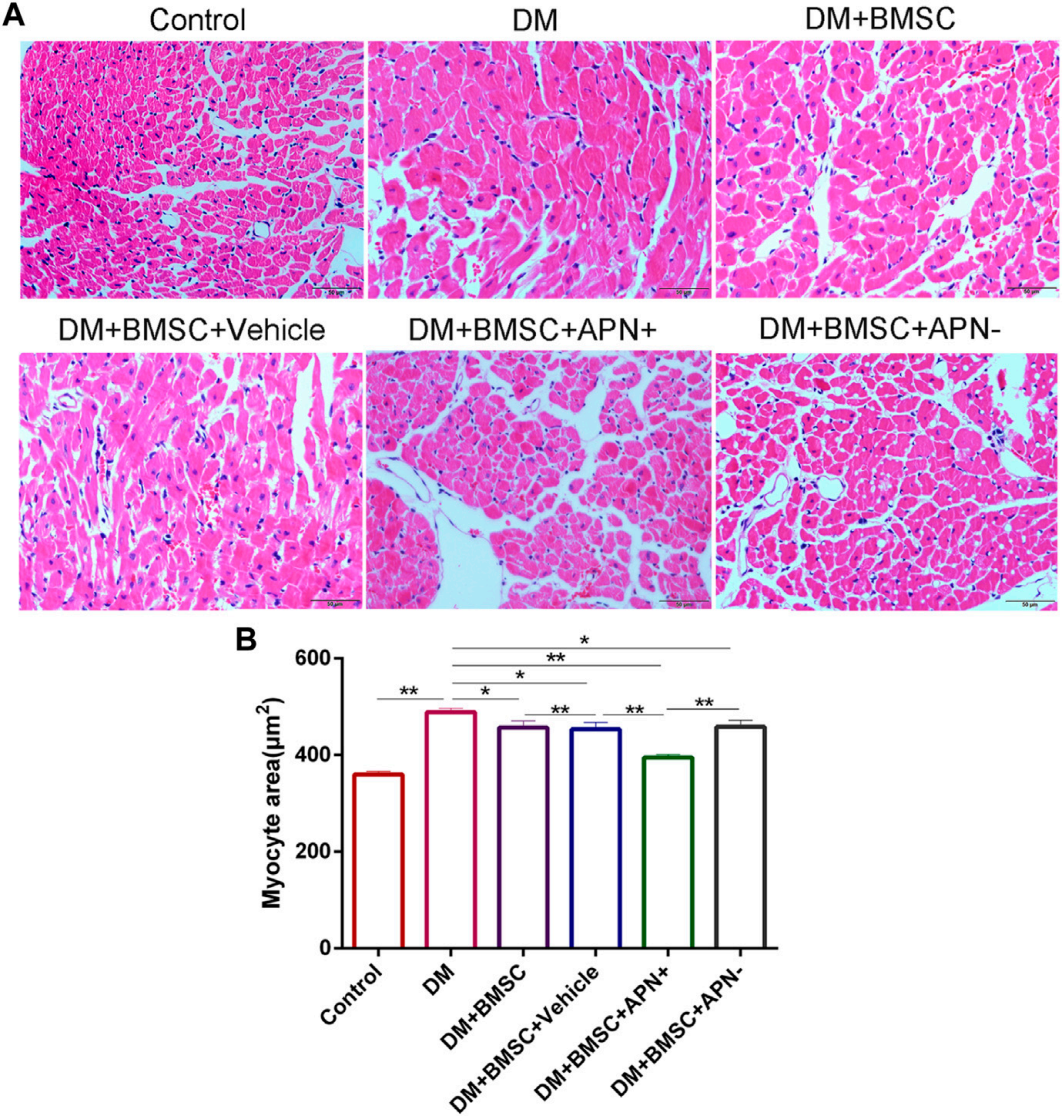
APN attenuated pathological changes in the hearts of diabetic rats. **(A)** Representative micrographs of myocardial tissue sections stained with hematoxylin and eosin (scale bar: 50 µm) **(B)** Quantitative analysis of myocyte size area. Data are mean ± SD; *p < 0.05, **p < 0.01.

**FIGURE 5:**
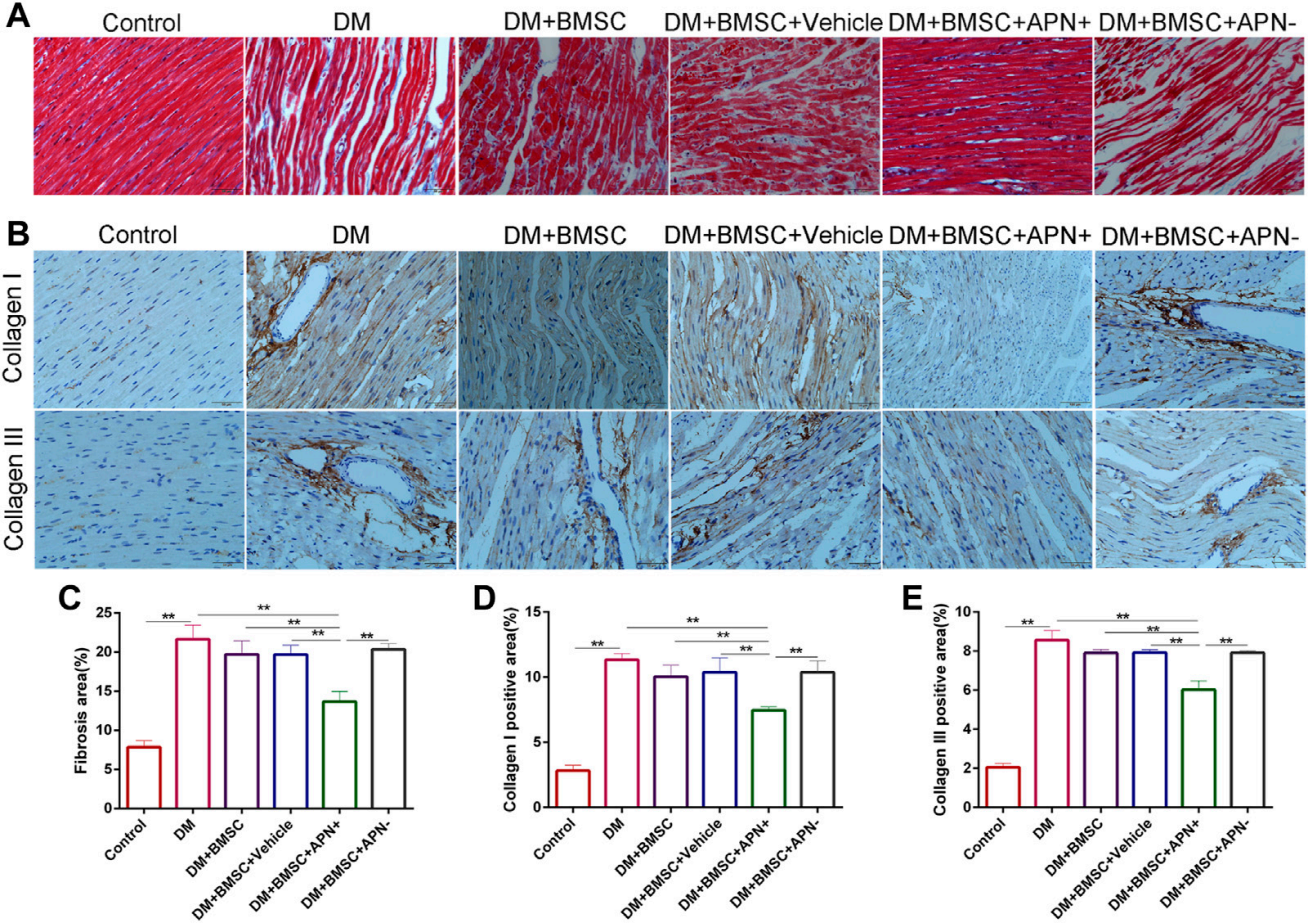
Effect of APN on collagen deposition. **(A)** Representative Masson’s trichrome staining **(B)** Immunostaining of collagen I and collagen III. **(C)** Quantitative analysis of Masson’s trichrome staining. **(D)** Immunohistochemistry analysis of collagens I. **(E)** Immunohistochemistry analysis of collagens III. Data are mean ± SD; *p < 0.05, **p < 0.01.

**FIGURE 6:**
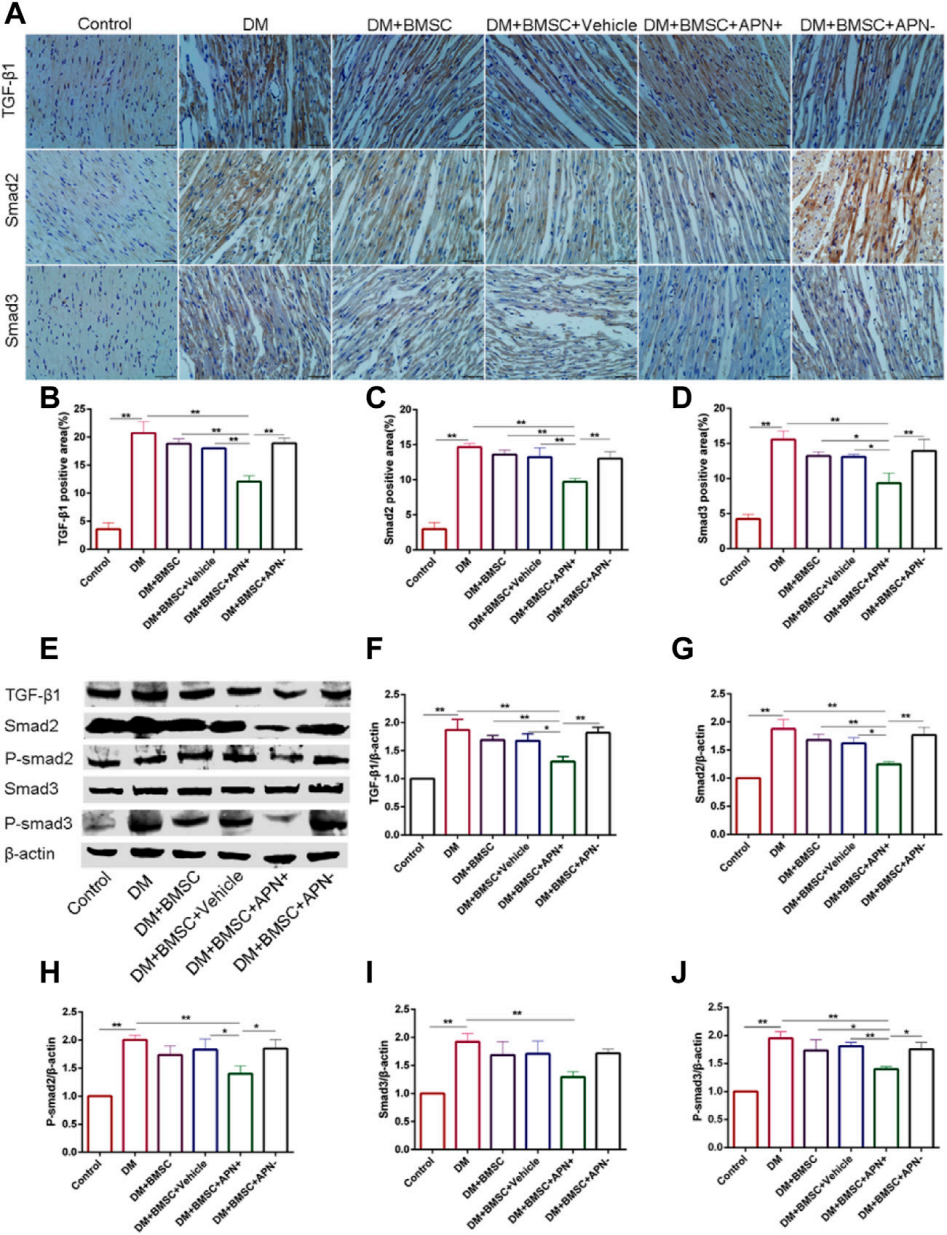
The expression of TGF-β1/Smad 2,3 in all groups rats. **(A)** Immunostaining of TGF-β1, Smad2 and Smad3. **(B)** Quantitative analysis of TGF-β1. **(C)** Quantitative analysis of Smad2. **(D)** Quantitative analysis of Smad3. **(E)** Representative Western blot: TGF-β1, Smad2, P-smad2, Smad3, and P-smad3. **(F)** The Western blot assay of TGF-β1. **(G)** The Western blot assay of Smad2. **(H)** The Western blot assay of P-smad2. **(I)** The Western blot assay of Smad3. **(J)** The Western blot assay of P-smad3. Data are mean ± SD; *p < 0.05, **p < 0.01.

The authors apologize for this error and state that this does not change the scientific conclusions of the article in any way. The original article has been updated.

